# Designing a digital tuberculosis treatment support and monitoring system for primary care: a user-centered qualitative study of patients, families, and healthcare workers

**DOI:** 10.1186/s12913-026-15005-z

**Published:** 2026-06-26

**Authors:** Agus Santosa, Neti Juniarti, Tuti Pahria, Raini Diah Susanti

**Affiliations:** 1 Nursing Doctoral Program, Faculty of Nursing, Universitas Padjadjaran, Sumedang, West Java Indonesia; 2 Department of Nursing, Faculty of Health Sciences, Universitas Muhammadiyah Purwokerto, Banyumas, Central Java Indonesia; 3 Department of Community Health Nursing, Faculty of Nursing, Universitas Padjadjaran, Sumedang, West Java Indonesia; 4 Department of Medical-Surgical Nursing, Faculty of Nursing, Universitas Padjadjaran, Sumedang, West Java Indonesia

**Keywords:** Tuberculosis, Digital health, Digital adherence technologies, Health information systems

## Abstract

**Background:**

Tuberculosis (TB) treatment requires prolonged adherence, continuous support, and coordinated monitoring within primary healthcare settings, where documentation, communication, and follow-up processes are often fragmented and resource-constrained. Although digital adherence technologies (DATs) have been introduced to support TB care, many systems have been developed with limited integration of stakeholder perspectives and routine healthcare workflows.

**Objective:**

This study aimed to explore user experiences, implementation challenges, and system requirements to inform the conceptual design of a digital tuberculosis treatment support and monitoring system for primary care.

**Methods:**

A qualitative study situated within a user-centered design framework was conducted in primary healthcare facilities in Banyumas District, Indonesia. Semi-structured interviews were conducted with 18 participants, including six TB patients, six family members, and six TB program nurses. Data were analyzed using thematic analysis. Identified user needs were subsequently translated into functional and non-functional system requirements to inform the conceptual system design.

**Results:**

Three interrelated themes emerged across stakeholder groups: maintaining TB treatment adherence as a prolonged and dynamic process, challenges in communication and coordination of family involvement in treatment support, and healthcare worker challenges in monitoring and care coordination. Cross-cutting needs included continuity of support, timely communication, shared visibility of treatment progress, and workflow integration. These findings were translated into functional requirements, including configurable reminders, role-based communication, clinician dashboards, and follow-up documentation tools, as well as non-functional requirements related to usability, privacy, accessibility, and workflow alignment. A conceptual digital treatment support and monitoring system was subsequently developed to support coordinated, role-based care within primary healthcare settings.

**Conclusion:**

This study provides a structured, user-centered foundation for the conceptual design of a digital tuberculosis treatment support and monitoring system for primary care. By integrating multi-stakeholder perspectives, the proposed framework supports a coordinated approach to treatment support, adherence monitoring, and continuity of care. The systematic translation of qualitative findings into explicit system requirements also offers a transparent and replicable pathway for early-stage digital health development in tuberculosis and other chronic disease contexts.

**Supplementary Information:**

The online version contains supplementary material available at https://doi.org/10.1186/s12913-026-15005-z.

## Introduction

Tuberculosis (TB) remains a major global health challenge and a continuing priority for health systems worldwide. Recent reporting from the World Health Organization (WHO) indicates that TB continues to impose a substantial burden across many regions, particularly in low- and middle-income countries (LMICs). TB treatment is prolonged and requires sustained adherence to medication. Supporting sustained medication adherence and continuity of follow-up is often difficult in routine primary healthcare settings, where resources and time are limited [1, 2].

Standard approaches to TB treatment support and follow-up typically rely on clinic-based documentation and treatment supervision. These processes can be time-consuming and frequently depend on paper-based records, which may create gaps in continuity when patients move between services or when documentation is incomplete. Workload constraints in primary care TB services have also been documented, particularly the time required for repeated patient visits and recording tasks [3–5]. Families often provide informal support for patients undergoing treatment; however, such support is rarely structured or integrated with routine treatment support and care coordination processes [6].

To address these challenges, WHO and international partners have promoted digital health as a strategy to strengthen TB prevention and care [7]. Digital adherence technologies (DATs), including SMS reminders, medication event monitoring systems, and video-supported treatment, have been introduced to support treatment adherence [8]. However, studies have shown that patient experience and acceptability vary across modalities, including between in-person directly observed therapy (DOT) and video-based approaches [9]. Furthermore, the implementation of digital systems often depends on infrastructure readiness, data management workflows, and governance arrangements, which can vary widely across LMIC settings [5, 10]. In many settings, digital interventions remain focused primarily on adherence tracking, with less attention to relational support, family involvement, workflow integration, and continuity of care within primary healthcare systems.

Effective digital tools must therefore align with users’ needs and the realities of healthcare settings. User-centered and participatory design approaches are increasingly used to improve the usability and adoption of digital health technologies [11, 12]. Evidence across digital health research suggests that factors such as digital literacy, interface design, and accessibility strongly influence user engagement and sustained use. Practical frameworks and co-design methods have also been proposed to support human-centered digital health development in settings with emerging digital health ecosystems [13–16], potentially facilitating better integration of digital tools into existing primary healthcare workflows and support systems.

Existing studies on digital tuberculosis interventions have primarily focused on treatment outcomes, adherence effectiveness, or implementation barriers rather than early-stage user-centered requirement elicitation and collaborative system design [8–10]. In addition, studies integrating perspectives from patients, family members, and frontline healthcare workers within primary healthcare contexts remain limited, particularly in LMIC settings. In Indonesia, evidence directly informing the design of digital tuberculosis treatment support and monitoring systems within primary healthcare settings remains scarce. A multi-stakeholder perspective is therefore needed to identify priority system features, acceptable workflows, and implementation constraints relevant to primary care. Beyond informing digital system development, understanding these stakeholder perspectives may also support improvements in service delivery, care coordination, continuity of care, and adherence support.

This study represents the early user-centered design phase of an ongoing digital health development initiative, focusing on needs assessment, requirement elicitation, and conceptual system design. Therefore, this study aimed to explore user experiences, implementation challenges, and system requirements to inform the design of a digital tuberculosis treatment support and monitoring system for primary care.

## Methods

### Study design

This study employed a qualitative exploratory design situated within a user-centered design (UCD) framework. In the context of digital health development, UCD commonly involves iterative phases including needs assessment, requirement elicitation, conceptual design, prototyping, and usability evaluation. The present study focused on the early UCD phases of needs assessment and requirement elicitation to inform the conceptual design of a digital tuberculosis treatment support and monitoring system for primary care. Within this framework, thematic analysis was used to identify patterns across stakeholder experiences, while requirement elicitation principles guided the translation of identified user needs into functional and non-functional system requirements [17].

### Study setting

This study was conducted in Banyumas District, Central Java, Indonesia, within primary healthcare facilities responsible for TB treatment and monitoring. The district represents a mix of urban, peri-urban, and rural service areas, reflecting diverse healthcare access conditions and patient backgrounds.

### Participants and sampling

Participants were purposively recruited to capture diverse perspectives related to tuberculosis treatment support, adherence, and care coordination within primary healthcare settings. Consistent with the UCD framework, three key stakeholder groups involved in routine tuberculosis care were included: patients undergoing TB treatment, family members involved in caregiving, and healthcare workers. Sampling aimed to achieve variation in age, gender, and treatment duration among patients to capture a broad range of treatment experiences across the course of therapy. Variation was also sought in caregiving roles among family members and professional experience among healthcare workers.

Eligible patients were adults (≥ 18 years) currently receiving TB treatment and able to provide informed consent. Family members were individuals who actively supported patients during treatment, such as spouses, parents, children, or other household members. Healthcare workers in this study were TB program nurses directly responsible for treatment support, patient follow-up, adherence monitoring, and routine tuberculosis documentation within primary healthcare services.

A total of 18 participants were included: six patients, six family members, and six TB program nurses. The sample size was guided by qualitative research principles emphasizing depth of insight, contextual understanding, and variation in stakeholder experiences rather than statistical representation. Data collection continued until thematic saturation was reached within and across stakeholder groups [18].

### Data collection

Data were collected through semi-structured, in-depth interviews conducted with participants from each stakeholder group. Interview guides were developed based on tuberculosis treatment support and monitoring practices, primary healthcare workflows, and user-centered design principles. The guides were reviewed by the research team to ensure clarity, contextual relevance, and alignment with the study objectives. Questions explored daily treatment experiences, adherence support practices, communication and follow-up processes, caregiving roles, workflow challenges, and expectations regarding digital support systems.

Interviews were conducted face-to-face in private settings at primary healthcare facilities or participants’ homes, depending on participant preference. Each interview lasted approximately 30–60 min and was audio-recorded with participant consent. Field notes were taken to capture contextual observations and non-verbal cues.

Interviews were conducted in Bahasa Indonesia by the first author, a nursing academic with experience in tuberculosis care and qualitative research. The interviewer had no prior clinical relationship with participants. All recordings were transcribed verbatim in the original language and reviewed for accuracy prior to analysis.

### Data analysis and translation process

Interview transcripts were analyzed using thematic analysis following the six-step approach described by Braun and Clarke [19], which includes familiarization with the data, generation of initial codes, searching for themes, reviewing themes, defining and naming themes, and producing the report. Transcripts were managed and coded using NVivo software. Initial familiarization was conducted through repeated reading of transcripts to identify recurring experiences related to tuberculosis treatment support, adherence practices, communication processes, caregiving roles, and workflow challenges. Codes were generated inductively and subsequently organized into broader themes representing shared patterns across stakeholder groups.

To enhance analytical rigor, two researchers independently coded a subset of transcripts and discussed discrepancies until consensus was reached. The remaining transcripts were then coded using the agreed coding structure, with regular team discussions conducted to refine theme interpretation and ensure consistency across stakeholder perspectives. Following thematic analysis, the identified themes were synthesized into explicit user needs. In the context of this study, user needs referred to practical expectations, challenges, and support requirements expressed by participants regarding tuberculosis treatment support, monitoring, communication, and care coordination processes.

The study then proceeded to the requirement elicitation phase within the UCD framework. Requirement elicitation refers to the systematic process of translating identified user needs into system-level specifications that can inform digital system development [20]. During this phase, user needs were translated into functional and non-functional system requirements. Functional requirements referred to specific system capabilities and features necessary to support user activities, such as medication reminders, adherence monitoring, communication functions, and follow-up documentation. Non-functional requirements referred to system qualities and operational characteristics necessary to support usability and implementation feasibility, including privacy, accessibility, flexibility, workflow integration, and ease of use.

The identified requirements were subsequently mapped to core system components to inform the conceptual architecture of the proposed digital tuberculosis treatment support and monitoring system. Requirement translation decisions and system mapping processes were discussed iteratively within the research team to ensure alignment between qualitative findings, stakeholder needs, and system design interpretation.

### Trustworthiness and rigor

Trustworthiness was ensured by addressing credibility, dependability, confirmability, and transferability throughout the study [21]. Credibility was enhanced through triangulation across three stakeholder groups—patients, family members, and healthcare workers—allowing comparison of perspectives on monitoring practices.

Dependability and confirmability were supported through peer debriefing and regular team discussions during coding and requirement translation. An audit trail documenting coding decisions, theme development, and requirement mapping processes was maintained throughout the study.

Transferability was strengthened by providing detailed descriptions of the study setting and participant characteristics to enable contextual interpretation. Representative participant narratives were used to support theme development while maintaining confidentiality.

## Results

### Participant characteristics

A total of 18 participants were included, comprising six tuberculosis patients, six family members, and six healthcare workers. Among patients, four were male and two were female, with varied educational backgrounds and treatment durations. The mean age of patients was 45.3 ± 7.8 years. Family participants were predominantly female and primarily spouses or close relatives who lived with or closely supported patients during treatment. The mean age of family members was 47.0 ± 15.8 years. Healthcare workers were TB program nurses from six primary healthcare centers. Their mean age was 32.7 ± 6.8 years, and the average duration of professional experience managing TB programs was 6.3 ± 4.5 years. Detailed participant characteristics are provided in the Supplementary.

### User experiences and monitoring challenges

Thematic analysis identified interrelated stakeholder experiences associated with tuberculosis treatment support, adherence, communication, and care coordination within primary healthcare settings. Findings across patients, family members, and healthcare workers highlighted challenges in sustaining treatment continuity, coordinating monitoring activities, managing communication processes, and integrating support into routine care delivery.

#### Maintaining TB treatment adherence as a prolonged and dynamic process

Patients described maintaining tuberculosis treatment adherence as a prolonged and dynamic process involving medication-related physical discomfort, emotional adjustment, and continuous efforts to sustain medication routines throughout therapy. Participants recalled that the initial period following diagnosis was frequently characterized by uncertainty and denial, particularly when symptoms prior to diagnosis had been mild or nonspecific. One participant explained, *“At first*,* I did not believe the diagnosis because I did not feel any symptoms… I even repeated the examination three times to make sure”* (P4). Commonly reported side effects included nausea, fatigue, itching, and reduced appetite, which often disrupted daily routines and contributed to difficulties in sustaining regular medication intake. As described by one participant, *“After taking the medication*,* I immediately vomited… eventually I stopped taking the medicine for some time”* (P3).

Several participants reported that medication-related discomfort occasionally led to delayed or missed doses, particularly during the initial treatment period. In response, patients developed various coping strategies to maintain adherence, including establishing fixed medication schedules, setting alarms, and relying on reminders and encouragement from family members. Support and reassurance from healthcare workers were also described as important in helping patients understand side effects and continue treatment despite discomfort.

As treatment progressed, many patients reported gradual acceptance of their condition and increased motivation to complete therapy. Fear of relapse, desire for recovery, and personal responsibility emerged as important drivers of adherence. However, participants also described periods of treatment fatigue associated with the prolonged duration of therapy. One participant stated, *“I am already very tired of taking this medication because the treatment lasts for nine months”* (P1). These findings suggest that adherence was influenced not only by individual motivation but also by the availability of support, timely communication, and accessible treatment information.

Across patient narratives, sustained adherence depended on continuous support from family members and healthcare workers, accessible treatment information, and ongoing communication throughout prolonged tuberculosis treatment.

#### Challenges in structured communication and coordination of family involvement in TB treatment support

Family members described playing a central role in supporting treatment continuity throughout the tuberculosis care process. Their involvement extended beyond medication reminders and accompanying healthcare visits to include emotional encouragement, observation of side effects, and day-to-day supervision of treatment routines. One family member explained, *“Sometimes I reminded the patient to take the medication*,* prepared the water*,* and also prepared the medicine”* (F1). In many cases, family members functioned as informal treatment supporters despite having limited formal guidance regarding tuberculosis care and adherence monitoring. The extent of family involvement varied according to caregiving availability and household circumstances, with some families sharing responsibilities among multiple family members while others relied on a single primary caregiver. Family support was primarily sustained through household initiative and commitment rather than through a formally coordinated monitoring process.

Participants described caregiving as both emotionally and practically demanding. Family members frequently reported feelings of worry, uncertainty, and responsibility following the patient’s diagnosis, particularly during periods when patients experienced medication side effects or reduced motivation to continue treatment. Although participants described receiving information and guidance from healthcare workers during clinic visits, ongoing communication and support were largely informal and depended on individual initiative. As one participant stated, *“We felt worried*,* afraid*,* and tired*,* but we had to be patient”* (F2). These responsibilities were often balanced alongside work obligations and household duties, creating challenges in maintaining consistent monitoring and support.

Communication with healthcare workers was commonly conducted during clinic visits or through WhatsApp messaging when family members needed information about treatment-related concerns, or follow-up care. However, communication was largely initiated by families and was not supported by structured mechanisms for routinely sharing information about treatment progress or adherence status. One participant expressed the need for clearer, more continuous information about the patient’s condition, stating that *“it would be helpful if the application could show whether the patient’s condition was improving”* (F2).

Across family narratives, treatment support was shaped by coordination challenges, limited access to structured information, and inconsistent communication pathways. These findings underscore the importance of digital systems that improve treatment visibility, facilitate communication, and support family involvement in adherence monitoring.

#### Healthcare worker challenges in monitoring and care coordination

Healthcare workers, particularly TB program nurses responsible for treatment follow-up and adherence monitoring, described tuberculosis treatment monitoring as a complex, time-intensive process involving adherence supervision, follow-up coordination, routine patient and family education, and documentation activities. Monitoring practices relied heavily on paper-based documentation and in-person follow-up. As one healthcare worker stated, *“Yes*,* everything is still manual”* (H1), which often created challenges in maintaining information continuity across treatment phases.

Participants reported that treatment monitoring relied largely on individual initiative and informal communication rather than integrated systems. Messaging applications such as WhatsApp were commonly used to contact patients and family members; however, these approaches were described as fragmented and difficult to coordinate across multiple patients. Monitoring practices also remained highly dependent on trust-based reporting. One participant explained, *“We often relied on mutual trust”* (H4), while another stated, *“We do not really know whether the medication is actually taken at home”* (H6). Healthcare workers further noted difficulties in identifying missed doses, treatment interruptions, or emerging side effects in a timely manner, particularly when communication was inconsistent or patients missed scheduled visits.

Administrative workload was a major challenge to effective monitoring. In addition to patient follow-up responsibilities, healthcare workers were required to complete routine reporting and documentation tasks, often resulting in duplicated recording processes and increased time burden. One participant explained, *“There are many patients*,* but only a small number of staff”* (H5). Participants emphasized that any future digital system would need to support monitoring and communication more efficiently without introducing additional documentation complexity or disrupting existing workflows.

Across healthcare worker narratives, tuberculosis treatment monitoring involved multiple actors, including TB program nurses, family members, community health volunteers, and referral facilities. Nurses were responsible for patient follow-up, education, documentation, and program reporting, while families and community health volunteers supported day-to-day adherence monitoring. However, coordination among these actors relied largely on manual documentation, informal communication, and fragmented reporting mechanisms. As a result, healthcare workers reported limited real-time visibility of adherence status, difficulties identifying treatment interruptions, and workflow inefficiencies that hindered continuity of care. These findings illustrate how gaps in coordination and information flow may affect the effectiveness of tuberculosis treatment support within primary healthcare settings.

#### Cross-cutting design implications

Across stakeholder groups, common challenges included fragmented communication, inconsistent monitoring practices, limited visibility of treatment progress, and difficulties coordinating adherence support. These findings informed the identification of stakeholder needs and the development of functional and non-functional system requirements for the proposed digital treatment support and monitoring system.

### Stakeholder needs for digital tuberculosis treatment support and monitoring

Thematic synthesis of stakeholder narratives identified a range of stakeholder needs related to tuberculosis treatment support, monitoring, communication, and care coordination. These needs reflected the practical expectations, challenges, and contextual realities experienced by patients, family members, and healthcare workers throughout the treatment process. While perspectives differed across stakeholder groups, several shared needs emerged across the continuum of care. A summary of stakeholder needs is presented in Table 1.

#### Patients’ need for accessible and continuous treatment support

Patients expressed the need for structured support to maintain consistent medication intake throughout prolonged treatment. Participants highlighted the importance of timely reminders, ongoing encouragement, and support mechanisms that could be easily integrated into their daily routines. One participant explained that reminders from healthcare workers helped maintain treatment continuity, stating, *“When the medication was running out*,* the healthcare staff immediately contacted me through WhatsApp”* (P6).

Participants also emphasized the need for clear and accessible information regarding medication side effects and treatment progress. Patients valued explanations that could help them understand whether symptoms such as nausea, itching, fatigue, or reduced appetite were expected treatment effects and how their condition was progressing during therapy. They also valued timely reassurance and understandable explanations that could help them manage treatment-related concerns and maintain confidence in continuing treatment. Several participants preferred support mechanisms that were practical, easy to access, and compatible with their everyday activities.

#### Families’ need for coordinated communication and treatment visibility

Family members expressed the need for clearer guidance and support in assisting patients throughout tuberculosis treatment. Participants highlighted the importance of receiving practical information that could help them monitor treatment, respond to patient concerns, and provide ongoing support during prolonged therapy. Several participants also emphasized the need for clearer information regarding treatment progress. One family member stated, “If *possible*,* the application should include a feature showing whether the patient’s condition is improving”* (F2).

Participants further expressed the need for coordinated communication with healthcare workers and easier access to treatment-related information. Families valued communication channels that could facilitate clarification, provide timely updates, and support continuity of care. As one participant explained, *“I usually message the doctor when the medication is almost finished”* (F6). Families also highlighted the importance of features such as medication reminders, follow-up notifications, and treatment progress displays to support adherence monitoring more effectively. One participant noted that *“Features showing treatment progress are important so we can monitor the patient’s condition”* (F3).

#### Healthcare workers’ need for integrated and efficient TB monitoring systems

Healthcare workers expressed the need for integrated and efficient systems to support adherence monitoring, follow-up coordination, and communication with patients. Participants emphasized the importance of having timely access to adherence information, treatment updates, and patient-reported concerns to support more responsive follow-up and continuity of care. This need was reflected in participants’ desire for more objective and reliable monitoring mechanisms beyond reliance on patient self-report. As one participant explained, *“We only know based on whether patients are honest or not”* (H6).

Participants also identified the need for digital features that could facilitate structured communication, symptom reporting, adherence tracking, and follow-up management without increasing administrative burden. They valued systems capable of streamlining monitoring activities while supporting routine documentation and reporting processes. One healthcare worker noted that such a system *“would really make monitoring much easier”* (H3).

#### Cross-cutting user needs

Across stakeholder groups, several shared needs were identified, including continuity of treatment support, timely communication, clear role expectations in supporting adherence, and flexible support mechanisms that accommodate patients’ daily circumstances. Participants emphasized the importance of coordinated engagement among patients, family members, and healthcare workers to sustain treatment adherence and continuity of care. These shared stakeholder needs provided the foundation for translating qualitative findings into functional and non-functional system requirements.

**Table 1:** Summary of user needs across stakeholder groups

Stakeholder	Core needs	Contextual focus
Patients	-Structured support for consistent medication intake; -Accessible information on side effects and treatment progress; -Privacy and discretion in receiving support	Maintaining adherence amid forgetfulness, fluctuating motivation, and stigma
Family members	-Clear guidance for caregiving; -Shared visibility of treatment routines; -Structured communication with healthcare workers	Supporting patients while managing uncertainty and competing responsibilities
Healthcare workers	-Systematic monitoring mechanisms; -Efficient follow-up coordination; -Aligned communication and documentation processes	Reducing workload while ensuring continuity of care
Cross-stakeholder	-Continuity of monitoring; -Timely communication; -Clarity of roles; -Adaptive support across treatment phases	Coordinated and collaborative treatment monitoring

### Translation of user needs into system requirements

The identified user needs were translated into system requirements to inform the conceptual design of a digital tuberculosis treatment support and monitoring system. This process aimed to ensure traceability between qualitative findings and system-level specifications, aligning with user-centered design principles. User needs were categorized into functional and non-functional system requirements.

#### Functional system requirements

Functional requirements referred to system capabilities and features necessary to address identified user needs and support treatment-related activities across stakeholder groups. A structured overview of the translation from user needs to functional system requirements is presented in Table 2.

Based on patient needs for structured adherence support, the system should provide configurable medication reminders aligned with individual schedules and treatment phases. To address informational uncertainty, the system should include accessible educational content regarding common side effects, treatment milestones, and guidance for managing discomfort.

To support family involvement, the system should enable role-based access, allowing authorized family members to view adherence status and receive relevant updates with patient consent. Mechanisms for structured communication between patients, families, and healthcare workers should be incorporated to facilitate timely reporting of concerns and feedback.

To meet healthcare workers’ needs for systematic monitoring, the system should include clinician-facing dashboards that summarize adherence patterns, alerts for missed doses, and tools to document follow-up actions. These features should support the efficient identification of patients who require additional attention.

**Table 2:** Translation of user needs into functional system requirements

User need domain	Stakeholder perspective	Functional system requirement
Structured adherence support	Patients	Configurable medication reminders aligned with individual schedules and treatment phases
Informational reassurance	Patients	Educational modules on side effects and treatment milestones
Discreet support access	Patients	User-controlled privacy settings and confidential notification features
Caregiving coordination	Family members	Role-based family access to adherence status (with patient consent)
Structured communication	Family members, Healthcare workers	Integrated communication channels with documentation capability
Systematic adherence monitoring	Healthcare workers	Clinician dashboard summarizing adherence patterns and alerts for missed doses
Follow-up documentation	Healthcare workers	Structured follow-up recording tools linked to patient adherence data
Timely multi-party interaction	Cross-stakeholder	Unified messaging system connecting patients, families, and providers

#### Non-functional system requirements

Non-functional requirements reflect system qualities necessary to ensure usability, feasibility, and sustainability within routine care settings. A summary of non-functional system requirements is presented in Table 3.

The system should maintain a simple and intuitive interface suitable for users with varying levels of digital literacy. Flexible configuration options should allow customization of reminder timing and notification preferences. Privacy and confidentiality should be ensured through role-based access controls and user-managed data sharing permissions.

To address contextual barriers identified by participants, the system should minimize data entry burden for healthcare workers, function reliably in low-bandwidth environments, and remain compatible with commonly used smartphones. The design should complement existing tuberculosis program workflows rather than introduce parallel documentation systems.

**Table 3:** Non-functional system requirements derived from user needs

Requirement domain	System-level specification
Usability	Intuitive and simplified interface accommodating users with varying digital literacy levels
Flexibility	Configurable reminder timing and adaptable notification preferences
Privacy and confidentiality	Role-based access control and user-managed data sharing permissions
System efficiency	Minimal additional data entry burden for healthcare workers; streamlined workflow alignment
Accessibility	Compatibility with commonly used Android smartphones and functionality in low-bandwidth environments
Integration	Alignment with existing tuberculosis program workflows and documentation practices

### Conceptual design of the digital treatment support and monitoring system

**Fig. 1:**
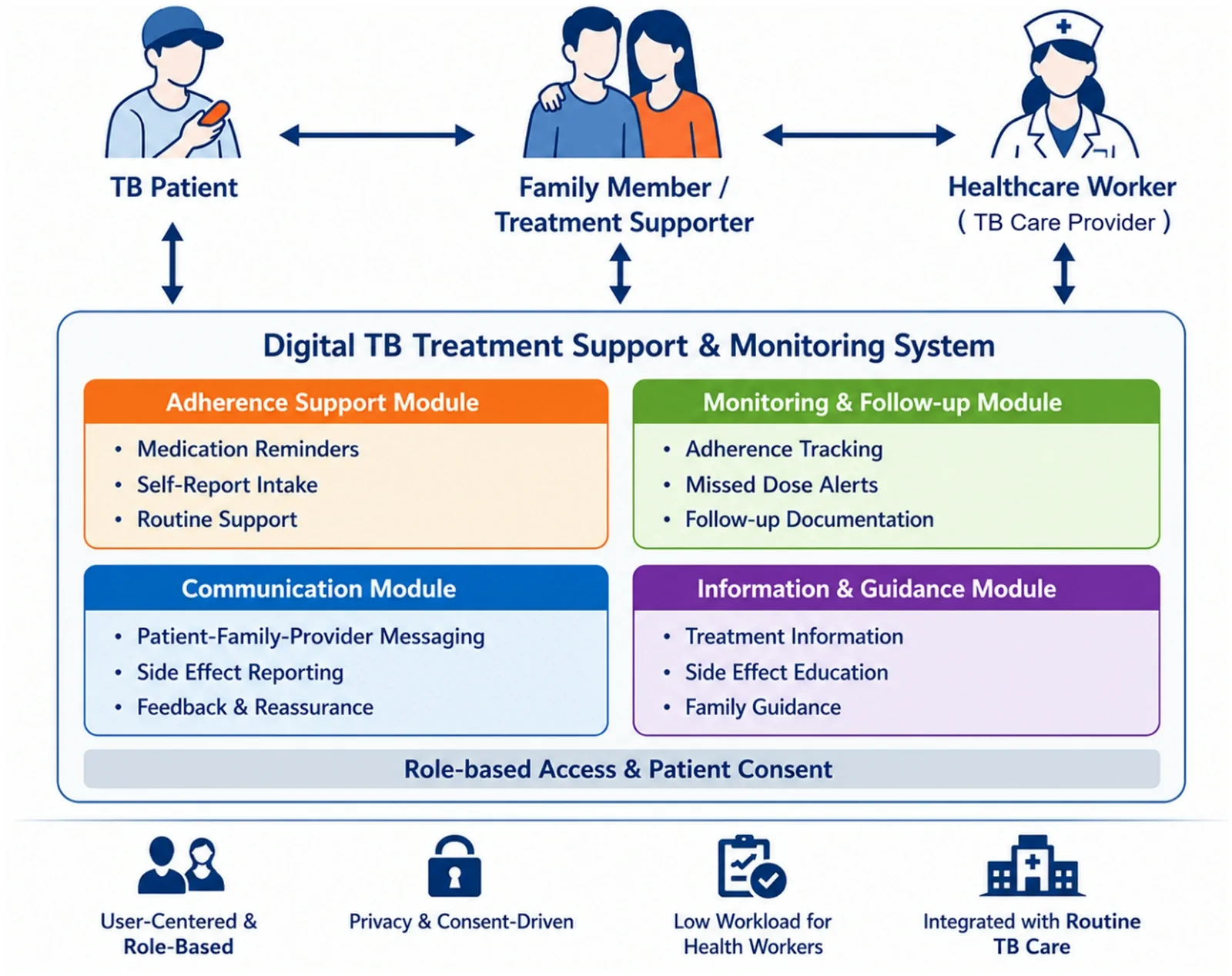
Conceptual design of the digital tuberculosis treatment support and monitoring system. This figure illustrates the conceptual framework derived from user needs identified among patients, family members, and healthcare workers. The system integrates adherence support, communication, monitoring, documentation, and care coordination modules within a shared digital environment, enabling role-based interaction and bidirectional information flow to support continuity of care and sustained treatment adherence

The conceptual design of the digital tuberculosis treatment support and monitoring system was developed based on empirically identified user needs from patients, family members, and healthcare workers. The proposed architecture integrates both functional and non-functional requirements identified in the previous section, ensuring traceability between qualitative findings and system-level design specifications. Rather than representing a finalized technical architecture, the conceptual model illustrates how treatment support, adherence monitoring, communication, documentation, and care coordination processes can be integrated within a shared digital environment (Fig. 1).

The system is designed around the patient as the primary actor in daily treatment support and self-reporting activities. Surrounding the patient are structured system components, including an adherence support module, educational and guidance resources, a role-based communication interface, and a clinician-facing monitoring dashboard. These components enable bidirectional information flow among patients, family members, and healthcare workers while maintaining differentiated access according to user roles.

The conceptual design emphasizes shared responsibility, structured coordination, and continuous feedback loops across stakeholders. By aligning treatment support and monitoring workflows with existing primary healthcare practices and accommodating contextual constraints such as digital literacy and connectivity limitations, the model supports the feasibility and continuity of care within routine tuberculosis services.

## Discussion

This study provides a user-centered foundation for developing a digital TB treatment support and monitoring system by systematically translating stakeholder experiences into structured system requirements. While DATs such as SMS reminders, medication event monitoring systems, and video-supported treatment have been promoted to strengthen adherence, their effectiveness and acceptability vary across contexts and modalities [8, 22].

Many digital solutions have been developed with limited early-stage integration of user perspectives, despite WHO guidance emphasizing context-sensitive implementation [3, 7]. By grounding system logic in the lived experiences of patients, families, and healthcare workers, this study addresses a gap between behavioral insight and digital system design.

The findings demonstrate that tuberculosis treatment monitoring is not an individual task but a distributed process involving interconnected actors. Patients experience fluctuating motivation and disruptions to daily routines; families provide informal yet essential caregiving support [6]; and healthcare workers operate within fragmented documentation and follow-up systems. These findings are consistent with prior reports describing workload constraints and structural limitations within primary TB care services [4, 5].

Such interdependence aligns with socio-technical perspectives in digital health, which emphasize that effective systems must integrate human workflows, communication patterns, and organizational realities rather than rely solely on technological automation. Unlike technology-driven solutions that prioritize automated alerts, the present findings suggest that reminder mechanisms must be embedded within broader communication, feedback, and shared-visibility structures. This systems-oriented translation strengthens the conceptual coherence between identified user needs and the proposed digital architecture [23].

An important contribution of this study is the explicit integration of family members into the digital monitoring model. Although family involvement has long been recognized as influencing adherence outcomes [6], many DAT implementations operationalize adherence as an individual behavior rather than a relational process [8, 21]. By incorporating role-based family access and structured visibility into caregiving, the proposed design advances a relationally integrated framework for digital treatment support and adherence monitoring.

Such an approach may be particularly relevant in LMICs settings, where household-based support structures are central to chronic disease management and where digital health implementation depends on contextual adaptation [5, 9]. Embedding relational dynamics into digital architecture may enhance continuity of support and reduce treatment fatigue during prolonged therapy [24].

Healthcare worker narratives highlighted concerns regarding workload and fragmented monitoring systems. Evidence from TB service delivery demonstrates that documentation and follow-up tasks already impose substantial demands on primary care providers [4, 5]. Digital health interventions may fail when they introduce additional documentation burdens or disrupt established workflows [9].

The conceptual design presented in this study prioritizes summarized dashboards, structured follow-up logic, and minimal data-entry requirements to reduce digital overload. This approach is consistent with the broader digital health literature, which emphasizes usability, workflow alignment, and implementation readiness as determinants of sustained adoption [12–15].

By aligning digital functionality with existing TB program workflows rather than creating parallel systems, the proposed model increases the likelihood of real-world feasibility. This workflow-centered design contributes to implementation science by demonstrating how early qualitative assessment can inform scalable digital architecture prior to technical development.

This study contributes to the digital health literature by illustrating a structured pathway from qualitative needs assessment to conceptual system design in the context of tuberculosis care. Human-centered and participatory design approaches have been recommended to improve the usability and adoption of digital health technologies [10, 11, 16], particularly in settings with emerging digital ecosystems [25].

Rather than adopting a technology-first model, the present work applies a needs-driven strategy grounded in user experience, contextual constraints, and documented variability in digital literacy and access [12, 14]. This structured translation model provides methodological guidance for early-stage digital health development in chronic disease management contexts beyond tuberculosis.

By emphasizing shared responsibility, adaptive communication, role-based access, and usability considerations, the conceptual model addresses both behavioral and structural dimensions of adherence support. Although conducted within a single district in Indonesia, the core design principles identified—shared monitoring responsibility, relational inclusion, workflow alignment, and adaptive support mechanisms—are transferable to other TB-endemic settings with comparable primary healthcare infrastructures.

The structured progression from stakeholder experience to functional and non-functional system requirements provides a replicable framework for developing digital interventions. From a health services perspective, these findings highlight the importance of integrating digital tools within existing care delivery structures rather than introducing parallel systems. By aligning digital monitoring with routine workflows, role distribution, and communication practices, the proposed model has the potential to enhance service coordination, reduce monitoring gaps, and support continuity of care in primary healthcare settings. Future research should evaluate the usability, feasibility, and effectiveness of the proposed system in real-world implementation, including its impact on adherence outcomes and healthcare workflow efficiency.

This study has several strengths. First, it incorporates perspectives from three key stakeholder groups—patients, family members, and healthcare workers—providing a comprehensive foundation for digital system design. Second, it follows a structured user-centered approach, translating qualitative findings into explicit functional and non-functional system requirements. This systematic progression enhances transparency and reproducibility in early-stage digital health development.

However, several limitations should be acknowledged. The study was conducted in a single district in Indonesia, which may limit contextual generalizability. Additionally, system requirements were derived from qualitative synthesis and have not yet been validated through participatory prototyping, usability testing, or implementation trials. As with most qualitative studies, findings are context-dependent and shaped by participant perspectives. Future studies should assess usability, acceptability, implementation outcomes, and the system’s impact on adherence and workflow efficiency across a broader range of healthcare settings.

## Conclusion

This study provides a structured, user-centered foundation for the conceptual design of a digital tuberculosis treatment support and monitoring system for primary care. By integrating perspectives from patients, family members, and healthcare workers, the proposed model advances a coordinated and relational approach to digital treatment support and adherence monitoring, emphasizing shared responsibility, communication, and workflow alignment.

The systematic translation of stakeholder experiences into explicit functional and non-functional system requirements offers a transparent and replicable framework for early-stage digital health development in tuberculosis and other chronic disease contexts. The findings also highlight the importance of integrating digital tools within existing primary healthcare workflows to support continuity of care and coordinated treatment management. Future research should evaluate the usability, implementation feasibility, and effectiveness of the proposed system in routine healthcare settings.

## Supplementary Information

Below is the link to the electronic supplementary material.

Supplementary Material 1

## Data Availability

The datasets generated and analyzed during the current study are not publicly available due to the qualitative nature of the data and the need to protect participant confidentiality. De-identified data may be available from the corresponding author upon reasonable request.

## Abbreviations

DATs: Digital adherence technologies
DOT: Directly observed therapy
LMICs: Low- and middle-income countries
TB: Tuberculosis
WHO: World Health Organization

## References

[1] World Health Organization. Global tuberculosis report 2025. Geneva: World Health Organization; 2025.

[2] World Health Organization. Tuberculosis: fact sheet. Geneva: World Health Organization; 2025.

[3] World Health Organization. Handbook for the use of digital technologies to support tuberculosis medication adherence. Geneva: World Health Organization; 2018.

[4] Blok L, van den Hof S, Mfinanga SG, et al. Measuring workload for tuberculosis service provision at primary care level: a methodology. Hum Resour Health. 2012;10:11. 10.1186/1478-4491-10-11. PMC3375184 22640406

[5] van der Heijden YF, Hughes J, Ruhwald M, et al. Overcoming limitations of tuberculosis information systems: researcher and clinician perspectives. Public Health Action. 2019;9(3):120–7. 10.5588/pha.19.0014. PMC6827492 31803584

[6] Alinaitwe B, Shariff NJ, Madhavi Boddupalli B. Treatment adherence and its association with family support among pulmonary tuberculosis patients in Jinja, Eastern Uganda. Sci Rep. 2025;15(1):11150. 10.1038/s41598-025-96260-8. PMC11961641 40169867

[7] World Health Organization. Digital health for the end TB strategy: an agenda for action. Geneva: World Health Organization. 2015. Available from: https://www.who.int/publications/i/item/WHO-HTM-TB-2015.21

[8] Santosa A, Juniarti N, Pahria T, et al. Digital adherence technology to improve medication adherence in tuberculosis patients: a systematic review and meta-analysis randomized control trials. npj Prim Care Respir Med. 2025;35(1):52. 10.1038/s41533-025-00457-3. PMC12638299 41271764

[9] Chen EC, Owaisi R, Goldschmidt L, et al. Patient perceptions of video directly observed therapy for tuberculosis: a systematic review. J Clin Tuberc Other Mycobact Dis. 2024;35:100406. 10.1016/j.jctube.2023.100406. PMC10877938 38380432

[10] Aboye GT, Simegn GL, Aerts JM. Assessment of the barriers and enablers of the use of mHealth systems in Sub-Saharan Africa according to the perceptions of patients, physicians, and health care executives in Ethiopia: qualitative study. J Med Internet Res. 2024;26:e50337. 10.2196/50337. PMC11007608 38536231

[11] Lee S, Lee Y, Lee S, Islam S, Kim S. Toward developing a standardized core set of outcome measures in mobile health interventions for tuberculosis management: systematic review. JMIR Mhealth Uhealth. 2019;7(2):e12385. 10.2196/12385. PMC6399569 30777847

[12] Thomas V, Kalidindi B, Waghmare A, Bhatia A, Raj T, Balsari S. The vinyasa tool for mHealth solutions: supporting human-centered design in nascent digital health ecosystems. JMIR Form Res. 2023;7:e45250. 10.2196/45250. PMC10580130 37607881

[13] Durmuş A. The influence of digital literacy on mHealth app usability: the mediating role of patient expertise. Digit Health. 2024;10:20552076241299061. 10.1177/20552076241299. PMC11590160 39600388

[14] Khowaja K, Al-Thani D. New checklist for the heuristic evaluation of mHealth apps (HE4EH): development and usability study. JMIR Mhealth Uhealth. 2020;8(10):e20353. 10.2196/20353. PMC7657716 33112252

[15] Yang S, Jin M, Kessel R, Warrier G, Thrul J, Lee M, et al. Understanding inequalities in mobile health utilization across phases: systematic review and meta-analysis. J Med Internet Res. 2025;27:e71349. 10.2196/71349. PMC12352709 40811740

[16] Dederichs M, Nitsch F, Apolinário-Hagen J. Piloting an innovative concept of e-mental health and mHealth workshops with medical students using a participatory co-design approach and app prototyping: case study. JMIR Med Educ. 2022;8(1):e32017. 10.2196/32017. PMC8787659 35006085

[17] Müller A, Schaaf J. User or patient/human or person? development of a practical framework for applying user-centered design to vulnerable populations for digital transformation in healthcare. Digit Health. 2025;11:20552076251375835. 10.1177/20552076251375835. PMC12417668 40933080

[18] Hennink M, Kaiser BN. Sample sizes for saturation in qualitative research: a systematic review of empirical tests. Soc Sci Med. 2022;292:114523. 10.1016/j.socscimed.2021.114523. 34785096

[19] Ahmed SK, Mohammed RA, Nashwan AJ, et al. Using thematic analysis in qualitative research. J Med Surg Public Health. 2025;6:100198. 10.1016/j.glmedi.2025.100198.

[20] Zowghi D, Coulin C. Requirements elicitation: a survey of techniques, approaches, and tools. In: Aurum A, Wohlin C, editors. Engineering and Managing Software Requirements. Berlin Heidelberg: Springer; 2005. pp. 19–46. 10.1007/3-540-28244-0_2.

[21] Nowell LS, Norris JM, White DE, Moules NJ. Thematic analysis: striving to meet the trustworthiness criteria. Int J Qual Methods. 2017;16(1):1609406917733847. 10.1177/1609406917733847.

[22] Ridho A, Alfian SD, van Boven JFM, et al. Digital health technologies to improve medication adherence and treatment outcomes in patients with tuberculosis: systematic review of randomized controlled trials. J Med Internet Res. 2022;24(2):e33062. 10.2196/33062. PMC8908199 35195534

[23] Griffin AC, Moyer MF, Anoshiravani A, Hornsey S, Sharp CD. A sociotechnical approach to defining clinical responsibilities for patient-generated health data. npj Digit Med. 2025;8:270. 10.1038/s41746-025-01680-5. PMC12069522 40355597

[24] Al Mahmud A, Joachim S, Jayaraman PP, et al. Digital health interventions to support chronic disease management: systematic scoping review. JMIR Mhealth Uhealth. 2026;14:e63742. 10.2196/63742. PMC12803440 41533959

[25] Brubacher LJ, Oga-Omenka C, Beggs B, et al. Profiling digital technologies used to support the tuberculosis care cascade and their implementation across high burden countries: a systematic scoping review. Digit Health. 2026;12:20552076251410991. 10.1177/20552076251410. PMC12819991 41573360

