# Supplementary material for: Designing a digital tuberculosis treatment support and monitoring system for primary care: a user-centered qualitative study of patients, families, and healthcare workers
Source: BMC Health Serv Res. 2026 Jun 26;26:1346. doi: 10.1186/s12913-026-15005-z (PMC13632315; doi:10.1186/s12913-026-15005-z)
Supplement: Supplementary file 1 [file 12913_2026_15005_MOESM1_ESM.pdf]

Supplementary 1. Participant characteristics

Characteristics of study participants (n = 18)

Characteristic	Patients (n = 6)	Family Members (n = 6)	Healthcare Workers (n = 6)
**Sex,** n (%)			
Male	4 (66.7)	1 (16.7)	2 (33.3)
Female	2 (33.3)	5 (83.3)	4 (66.7)
**Age**, years			
Mean ± SD	45.3 ± 7.8	47.0 ± 15.8	32.7 ± 6.8
Range	25–54	25–66	28–46
**Education level**, n (%)			
Primary school	3 (50.0)	2 (33.3)	—
Junior high school	0 (0.0)	2 (33.3)	—
Senior high school	2 (33.3)	2 (33.3)	—
Diploma/Bachelor	1 (16.7)	0 (0.0)	6 (100)
**Employment status**, n (%)			
Unemployed	3 (50.0)	2 (33.3)	—
Self-employed	3 (50.0)	4 (66.7)	—
**Treatment-related experience**			
TB treatment duration (months)	2–8	—	—
Living with a TB patient	—	5 (83.3)	—
Years managing the TB program	—	—	6.3 ± 4.5

Supplementary Appendix 1. Semi-structured interview guide for patients

Purpose:
To explore the experiences, challenges, and needs of patients with tuberculosis (TB) regarding treatment adherence and to identify requirements for a digital treatment support and monitoring application.

A. Patient Demographic Information

1. Name: ______
2. Age: ______
3. Sex: Male / Female
4. Duration of TB treatment (months): ______
5. Employment status: ______
6. Highest educational level: ______

B. Experiences and Challenges During TB Treatment

1. Can you describe your experience while undergoing TB treatment?
2. From a personal perspective, what factors most influence your treatment process?
3. From a family or social perspective, what factors influence your treatment?
4. From the perspective of medication or therapy, what aspects have been most disturbing or difficult for you?
5. How would you describe your experience with healthcare facilities and healthcare workers during treatment?

C. Needs Related to a Digital Application

1. In your opinion, could a mobile phone application help support your TB treatment?
2. If such an application existed, what features would you expect or find helpful?
3. What kind of application design or interface would be easiest for you to use?

D. Barriers and Suggestions for Application Development

1. What barriers or challenges do you think might arise if you were required to use an application during treatment?
2. What are your expectations or suggestions to ensure that this application could genuinely help support your treatment process?

Supplementary Appendix 2. Semi-structured interview guide for family members

Purpose:
To explore the experiences, roles, and challenges of family members in supporting patients with tuberculosis (TB) during treatment, and to identify digital application features that may assist families in monitoring and supporting treatment adherence.

A. Family Member Demographic Information

1. Name: ______
2. Age: ______
3. Sex: Male / Female
4. Relationship to the patient:
   ☐ Spouse
   ☐ Child
   ☐ Parent
   ☐ Sibling
   ☐ Other: ______
5. Highest educational level: ______
6. Employment status: ______
7. Living in the same household as the patient: ☐ Yes ☐ No
8. Duration of the patient's TB treatment: ______ months

B. Experiences and Roles in Supporting Patients with TB

1. How did you feel when you learned that your family member had TB?
2. Can you describe your experience in supporting a family member undergoing TB treatment?
3. To what extent are you involved in supporting the patient during treatment (e.g., reminding medication intake, accompanying healthcare visits, preparing daily needs)?
4. What challenges or difficulties has your family experienced while supporting the patient?

C. Factors Influencing Patient Treatment Adherence

1. In your opinion, what factors most influence the patient's ability to follow TB treatment regularly?
2. Has the patient ever forgotten or been reluctant to take medication? If yes, what were the usual reasons?
3. From the family perspective, what factors are most helpful in encouraging the patient to take medication regularly?
4. From the social or environmental perspective, are there factors that make the patient feel embarrassed, reluctant, or burdened during treatment?

5. How would you describe your experience interacting with healthcare workers or healthcare services during the patient’s treatment?

D. Needs Related to a Digital Application

1. In your opinion, could a mobile phone application help families monitor or support patients with TB?
2. If such an application existed, what features would be most needed by families (e.g., medication reminders, communication with healthcare workers, side-effect information, patient progress records)?
3. What kind of application design or interface would be easiest for family members to use (e.g., simple layout, images, notifications)?

E. Barriers and Suggestions for Application Development

1. In your opinion, what barriers or challenges might arise if family members were required to use such an application?
2. What are your expectations or suggestions to ensure that this digital application could genuinely help families support patients with TB during treatment?

Supplementary Appendix 3. Semi-structured interview guide for healthcare workers

Purpose:
To explore the experiences, challenges, and needs of healthcare workers in monitoring tuberculosis (TB) treatment adherence, and to identify digital application features that are appropriate for routine primary healthcare settings.

A. Demographic Information

1. Name: ______
2. Age: ______
3. Sex: Male / Female
4. Position:
   ☐ TB Program Nurse
   ☐ TB Officer
   ☐ TB Program Coordinator
   ☐ Other: ______
5. Duration of working with TB patients: ___ years
6. Primary healthcare center location: __________

B. Experiences and Challenges in Monitoring TB Treatment

1. Can you describe your experience in monitoring TB treatment?
2. What methods do you usually use to monitor TB treatment adherence?
3. From the patient perspective, what challenges do you most frequently observe during TB treatment?
4. From the family or social perspective, what factors commonly affect patients during treatment?
5. From the medication or therapy perspective, are there side effects that frequently interfere with patient adherence?
6. From the healthcare service perspective, what limitations or challenges do you face in supporting TB treatment monitoring?

C. Needs Related to a Digital Application

1. In your opinion, could a digital application help support monitoring of TB treatment adherence? Why?
2. If such an application were developed, what features would be most important?
3. How should the application be designed to ensure ease of use for both patients and healthcare workers?

D. Potential Implementation Barriers, Expectations, and Recommendations

1. In your area, are TB patients generally familiar with using mobile phones or digital applications?

2. What technical barriers or challenges might arise during implementation?
3. From the healthcare worker perspective, would the application help support your work or potentially increase workload?
4. What are your expectations and recommendations to ensure that this application would be genuinely useful for supporting TB patient monitoring and care?

Note: The interview guides were originally developed in Bahasa Indonesia and translated into English for reporting purposes.
